# *Brucella* spp. are facultative anaerobic bacteria under denitrifying conditions

**DOI:** 10.1128/spectrum.02767-23

**Published:** 2023-10-26

**Authors:** Luca Freddi, Jorge A. de la Garza-García, Sascha Al Dahouk, Alessandra Occhialini, Stephan Köhler

**Affiliations:** 1 Institut de Recherche en Infectiologie de Montpellier (IRIM), CNRS, University of Montpellier, INSERM, Montpellier, France; 2 German Federal Institute for Risk Assessment, Berlin, Germany; 3 German Environment Agency, Berlin, Germany; Institut National de Santé Publique du Québec, Sainte-Anne-de-Bellevue, Québec, Canada

**Keywords:** *Brucella*, anoxia, facultative anaerobic, denitrification, nitrate reductase, nitrite reductase, nitric oxide reductase, nitrous oxide reductase

## Abstract

Bacteria of the genus *Brucella* are responsible for human and animal brucellosis worldwide and comprise the well-characterized, so-called classical species belonging to the core clade, with a host range limited to mammals, and the novel atypical strains mainly considered as non-zoonotic to date, but able to colonize non-traditional hosts such as amphibians. Despite marked phenotypical differences between the two groups, *Brucella* species display a high degree of genome identity, including genes encoding reductases involved in the denitrification pathway, a respiratory process able to reduce nitrate to nitrogen under anoxic conditions. *Brucella* spp. have always been considered as strictly aerobic pathogens. Our study, however, describes for the first time that atypical *Brucella* spp. grow well under anoxia, but in a strictly nitrate-dependent manner, whereas classical species show a heterogeneous behavior. The rate of anaerobic growth of the atypical species *Brucella microti* was dependent on nitrate concentration, highlighting a more efficient denitrification pathway with rapid consumption of intermediate nitrite, whereas the classical species *Brucella suis* exhibited lower, nitrate concentration-independent growth coupled with nitrite accumulation. Transcriptional studies performed under anoxic conditions revealed a higher expression of genes participating in the last three denitrification steps in *B. microti*, as compared to *B. suis*, which correlates with the rapid and efficient consumption of nitrite in this atypical species and its rapid growth.

**IMPORTANCE:**

Respiration is a fundamental and complex process that bacteria use to produce energy. Despite aerobic respiration being the most common, some bacteria make use of a mode of respiration in the absence of oxygen, called anaerobic respiration, which can yield advantages in adaptation to various environmental conditions. Denitrification is part of this respiratory process ensuring higher respiratory flexibility under oxygen depletion. Here, we report for the first time the evidence of anaerobic growth of *Brucella* spp. under denitrifying conditions, which implies that this genus should be reconsidered as facultative anaerobic. Our study further describes that efficient denitrification is not equally found within the *Brucella* genus, with atypical species showing a greater ability to denitrify, correlated with higher expression of the genes involved, as compared to classical species.

## INTRODUCTION

Brucellae are non-motile Gram-negative bacteria causing brucellosis, a worldwide zoonosis of major public health concern (1, 2). *Brucella* infections regularly occur in wildlife and livestock, with occasional spillover to humans, most commonly infected by *Brucella abortus*, *Brucella melitensis,* and *Brucella suis* (1, 3). These species were first described decades ago and belong to the “core” clade (4, 5). The transmission routes most relevant for humans are consumption of unpasteurized dairy products, direct contact with infected animals, or inhalation of aerosolized bacterial suspensions. Human brucellosis is usually characterized by undulant fever and enlarged lymph nodes during the acute phase, progressing to chronic courses in untreated patients, where bacteria are able to persist within granulomatous lesions in microaerobic or anaerobic environment (6 – 8).

*Brucella* is generally considered as an intracellular, facultative extracellular pathogen able to colonize phagocytic cells to evade host’s adaptive immune system, multiply, and spread throughout the organism (9 – 11). Analysis of the intramacrophagic virulome of *B. suis* indirectly reveals an environment low in nutrients and oxygen to which the pathogen has obviously adapted (12). Although *Brucella* is considered as strictly aerobic genus (13 – 15), the capacity of adaptation to low-oxygen conditions is crucial in the process of cell infection, when the oxygen concentration is lower than in the extracellular environment (16). Various respiratory pathways, principally based on the *cbb*3-type cytochrome *c* oxidase and the cytochrome *bd* ubiquinol oxidase, are present in *B. suis*, contributing to its resistance to oxygen depletion and revealing a high respiratory flexibility (17 – 19). In addition, the presence of nitrate reductase has been described, raising the question of possible anoxybiotic growth of *Brucella* (20, 21). However, to the best of our knowledge, experimental data regarding the obligate aerobic or facultatively anaerobic growth of *Brucella* are not available.

Respiration is a fundamental process in all living cells, resulting in ATP production following electron transfer from low-redox-potential electron donors such as NADH to a high-redox-potential electron acceptor (O_2_) (22). In prokaryotes, enhanced respiratory flexibility allows the use of alternative electron acceptors, including nitrogen oxides, sulfate, and oxyanions, and contributes to their ability to colonize microaerobic or anaerobic environments. Denitrification is a respiratory process in which nitrate (NO_3_) and nitrite (NO_2_) are reduced into gaseous nitric oxide (NO), nitrous oxide (N_2_O), and nitrogen (N_2_) under oxygen-limited conditions (23). *B. suis* and *B. melitensis* possess the four reductases Nar (NO_3_ reductase), Nir (NO_2_ reductase), Nor (NO reductase), and Nos (N_2_O reductase) needed to catalyze the complete denitrification cascade (23). Denitrification can provide energy for bacterial metabolism in oxygen-poor and/or anaerobic environments, allowing pathogenic bacteria such as *Brucella*, *Neisseria gonorrhoea* (24), and *Mycobacterium bovis* (25) to persist within the host. Denitrification provides these bacteria with an additional defense mechanism against NO, produced by macrophages to kill invading microorganisms (19, 26). The regulation of denitrification genes in *Brucella* occurs through Fnr-Crp proteins, which primarily function as positive transcription factors (27). In *B. melitensis*, two of these regulators, NarR and NnrA, control genes encoding Nar, and Nir, Nor, Nos, respectively (28). In *B. abortus*, activity of NtrYX as a redox sensor two-component system involved in oxygen sensing and regulation of the denitrification enzymes was described (29, 30). Moreover, the two-component system RegA/RegB plays a key role as a redox sensor in adaptation of *B. suis* to oxygen depletion and participates in transcriptional control of denitrification (31).

*Brucella* species are classified based on phylogeny and phenotypic properties, including host range preference (7, 32). In recent years, in addition to the above-mentioned six classical “core” species and the two species isolated from marine mammals, four new species have been described, expanding the ecology of the bacterium: *Brucella inopinata* from a human infection (33, 34), *Brucella microti* from voles (35), *Brucella papionis* from baboons (36), and *Brucella vulpis* from foxes (37). These novel and, at least some of them, atypical species, along with a growing number of *Brucella* sp. strains, are able to colonize hitherto unconventional hosts including non-mammalian vertebrates such as frogs (38 – 41) and fish (42). Most of them exhibit higher metabolic activity and faster growth rates than the classical core species, suggesting a metabolism better adapted to environmental survival (38). *B. microti* is associated with unexpected ecosystems because it persists in soil (43) and in aquatic environments (44). Despite its phylogenetic affiliation to the core clade of *Brucella* (4, 45) and a genome identity of 99.84% with *B. suis* 1330 (46). *B. microti* is phenotypically different from the classical core species and was therefore designated as “atypical” (5). Markedly higher rates of replication in broth and macrophage host cells indicate metabolic specificities that may be due to mutations or gene expression regulation, as demonstrated lately in the adaptation to acidic pH (47). Interestingly, *B. microti* is the first *Brucella* species described to be lethal in murine infection experiments (48), in addition to its capability to persist in environments outside the host. Using RNA-Seq analysis in the minimal medium at pH 4.5 and 7, we recently demonstrated species-specific acid resistance mechanisms in *B. microti*, including strong activation of the genes encoding denitrification enzymes (47). As the classical core *Brucella* species, the *B. microti* genome contains the *nar*, *nir*, *nor,* and *nos* gene clusters. Their increased expression in *B. microti* at low pH suggests a species-specific regulation of anaerobic/microaerobic respiration as a consequence of decreasing dissolved oxygen in acidic medium. Besides contributing to acid resistance, the maintenance of an active denitrification pathway in new species such as *B. microti* may be explained by denitrification in natural oxygen-deprived environments such as soil and water. Furthermore, the extensive use of nitrogen fertilizers leads to an accumulation of nitrates (49), which may promote survival and possibly proliferation of the bacteria.

In our study, we investigated the growth behavior and gene regulation of classical and novel *Brucella* species and strains under anaerobic conditions in the presence of alternative electron acceptors to get further insights on their oxygen requirement. Our results revealed that higher expression levels of denitrification genes in *B. microti* correlated with normal growth of atypical *Brucella* species in the presence of nitrates under anaerobic conditions, which is in contrast to the general assumption that the genus *Brucella* is strictly aerobic.

## MATERIALS AND METHODS

### Biosafety procedures

The *Brucella* strains were handled under BSL-3 conditions, according to French regulations. Inactivation of bacteria and DNA/RNA extractions were conducted in a class II biological safety cabinet (Thermo Scientific, USA).

### Bacterial strains and culture conditions

All *Brucella* species and strains (*B. suis* 1330 ATCC 23444, *B. microti* CCM 4915, *B. abortus* ATCC 23448, *B. melitensis* 16M ATCC 23456, *B. inopinata* BO1, *Brucella* sp. 83–210 from Australian rodents (50), and *Brucella* sp. 09RB8910 from African bullfrogs (38)) were grown in Tryptic Soy Broth (TSB) at 37°C with shaking. For the selection of mutants, kanamycin was added at a final concentration of 50 µg/ml.

To perform liquid cultures under anoxic atmosphere, 15 mL tubes were filled with TSB medium and autoclaved, covered with mineral oil, and closed with an air-tight screw cap until inoculation. *Brucella* strains and *Clostridium perfringens*, a bacterium with an obligate anaerobic metabolism, as control were suspended at 10^7^ CFU/ml and incubated both in the presence and absence of NaNO_3_ at 37°C without shaking. At each time point, one tube was sacrificed to measure the optical density at 600 nm (OD_600_) value, bacterial viability, and growth. Growth of the anaerobic *C. perfringens* was considered as an indicator for effective anoxia. To increase the number of samples cultivated under identical conditions, anoxic cultures were also conducted in air-tight jars containing GENbag anaer generator bags (BioMérieux, France). A 25 cm^2^ plastic cell culture flasks with vented caps, filled with freshly boiled TSB and inoculated at 10^7^ CFU/ml in the presence or absence of different concentrations of NaNO_3_, were placed into the jars. Anoxic conditions were systematically verified using Anaer Indicator strips (BioMérieux, France). Finally, to test the ability to form bacterial colonies, *Brucella* spp. were inoculated at 10^3^ CFU/ml on solid Tryptic Soy agar (TSA) plates in the presence of 10 mM NaNO_3_, placed in a jar with GENbag anaer generator bags, and incubated at 37°C for 10 days.

All bacterial growth experiments under aerobic or anoxic conditions were performed in triplicate. Bacterial viability was determined by plating serial dilutions of bacterial suspensions onto TSA and incubation at 37°C under standard aerobic conditions.

### RNA isolation and quantitative reverse transcriptase PCR (RT-qPCR)

After inoculation with overnight cultures at a concentration of 10^7^ CFU/ml in the presence of 50 mM NaNO_3_, triplicates of bacterial cultures of *B. microti* CCM 4915 and *B. suis* 1330 were placed into air-tight jars containing GENbag anaer generator bags (BioMérieux, France) and incubated in 25 cm^2^ plastic cell culture flasks at 37°C with shaking. At the corresponding time points, aliquots of the cultures were instantly inactivated by adding 1/10 volume of 30% phenol/ethanol solution and vigorous mixing, followed by centrifugation at 10,000 rpm and storage of the *Brucella* pellets at −80°C. Total RNAs were isolated using the mirVana RNA isolation kit (Ambion) according to the manufacturer’s instructions and were treated with Turbo RNase-free DNase (Ambion) to remove any residual DNA. Prior to reverse transcription, each RNA sample was tested by polymerase chain reaction (PCR) for residual DNA contamination and treated with DNase a second time if necessary. The RNA samples were quality-checked using an Agilent Bioanalyzer 2000.

Candidate genes representative of the denitrification pathway, as well as the 16S rRNA gene as reference, were selected, and their expression over time was studied by RT-qPCR, as described by de la Garza-Garcia et al. (47). Briefly, 1 µg of total RNA was randomly reverse-transcribed (hexamers) into cDNA in a final reaction volume of 20 µL using SuperScript VILO Master Mix at 42°C for 90 min. Following 1:20 dilution of cDNA for candidate genes and 1:2000 dilution for the 16S rRNA gene, cDNA samples were amplified in triplicates using Syber Green I Master (Roche) in a final volume of 1.5 µL per reaction and a LightCycler 480 (Roche). Primers were designed with the Primer3 software (Table 1). The 396-well microplates were prepared using an Echo 525 Liquid Handler (Labcyte Inc.) at the Montpellier GenomiX (MGX) platform. The relative fold change of gene expression was calculated by the ΔΔCt method based on the normalized threshold cycles Ct (51).

**TABLE 1:** Primers selected for RT-qPCR validation

Gene ID		Encoded protein	Primers	
			For 5'- > 3'	Rev 5'- > 3'
*B. suis*	*B. microti*			
BRA0298	BMI_II292	NarH	CATCCTCGCCAAGATTTTCG	CCATTCGATCTTCTCCATCC
BRA0260	BMI_II254	NirK	GAAAGTGGAGCTGGTCGATC	CCGCATCATCAATGACGATC
BRA0249	BMI_II243	NorB	GGAGCTTTACAGCACCAAGC	TGAAGAAGGGCTGTTCAAGG
BRA0274	BMI_II269	NosR	AAGGGGAACGAGCTTCTAGG	TTGGTATCAATGCCCACAAC

### Construction of a *B. microti narG* deletion mutant

Inactivation of *narG* encoding the α-subunit of the NO_3_-reductase in *B. microti* was achieved by homologous recombination using plasmid pUC18 containing a deleted copy of *narG*, in which a 770 bp *NcoI*-fragment was replaced by a kanamycin resistance cassette, as described earlier for the construction of a *B. suis narG*-mutant (19).

### Nitrate and nitrite measurements

The utilization of nitrogen oxides by the bacteria during growth in broth supplemented with nitrates was assessed by measuring NO_3_ and NO_2_ concentrations in the medium using the Griess reagent, modified from a previous protocol (52). Briefly, 100 µL of the collected supernatants were dispensed in 96-well microplates and mixed with 25 µL of 1% sulfanilic acid in 30% acetic acid and 25 µL of 2% dimethyl-1-naphthylamine in 60% acetic acid. Calibration curves were established with serial dilutions of NaNO_2_ in the µM range, and optical densities were measured at 590 nm using a microplate reader (Tecan Sunrise). For the measurement of residual NO_3_ concentrations in culture supernatants, zinc powder was added to the assay as catalyst for transformation into NO_2_. In this case, a calibration curve was obtained starting from defined concentrations of NaNO_3_ in the presence of zinc.

### Statistical analysis

Statistical analyses were applied to assess the effect of nitrate on anaerobic growth of *Brucella* species and strains. To state about significance at given time points in the differences of growth between individual strains under anoxic conditions in the presence of nitrates, or of growth of a given strain under anoxia in the absence or presence of nitrates, the log CFU/ml values were analyzed. Evaluation of the significance of the differences between *Brucella* strains in the use of nitrates was performed by comparing the nitrite mean concentration values (mM). In both cases, the student *t*-test was applied, and 95% confidence intervals were established. *P*-values ≤0.05 were considered significant.

## RESULTS

### *Brucella suis* 1330 and *Brucella microti* CCM4915 grow under anaerobic conditions in the presence of nitrates

Since the genes encoding the reductases Nar, Nir, Nor, and Nos responsible for the four denitrification steps are intact, and the corresponding proteins are almost identical in *B. suis* 1330 and *B. microti* CCM4915 (data not shown), we investigated whether *Brucella* can grow anaerobically in the presence of nitrate as the sole electron acceptor. Two experimental setups allowed us to assess *Brucella* growth capacities under anaerobic conditions in TSB: (1) Culture tubes containing medium overlaid with mineral oil; (2) Culture flasks in air-tight jars containing anaer GENbags.

**Fig 1:**
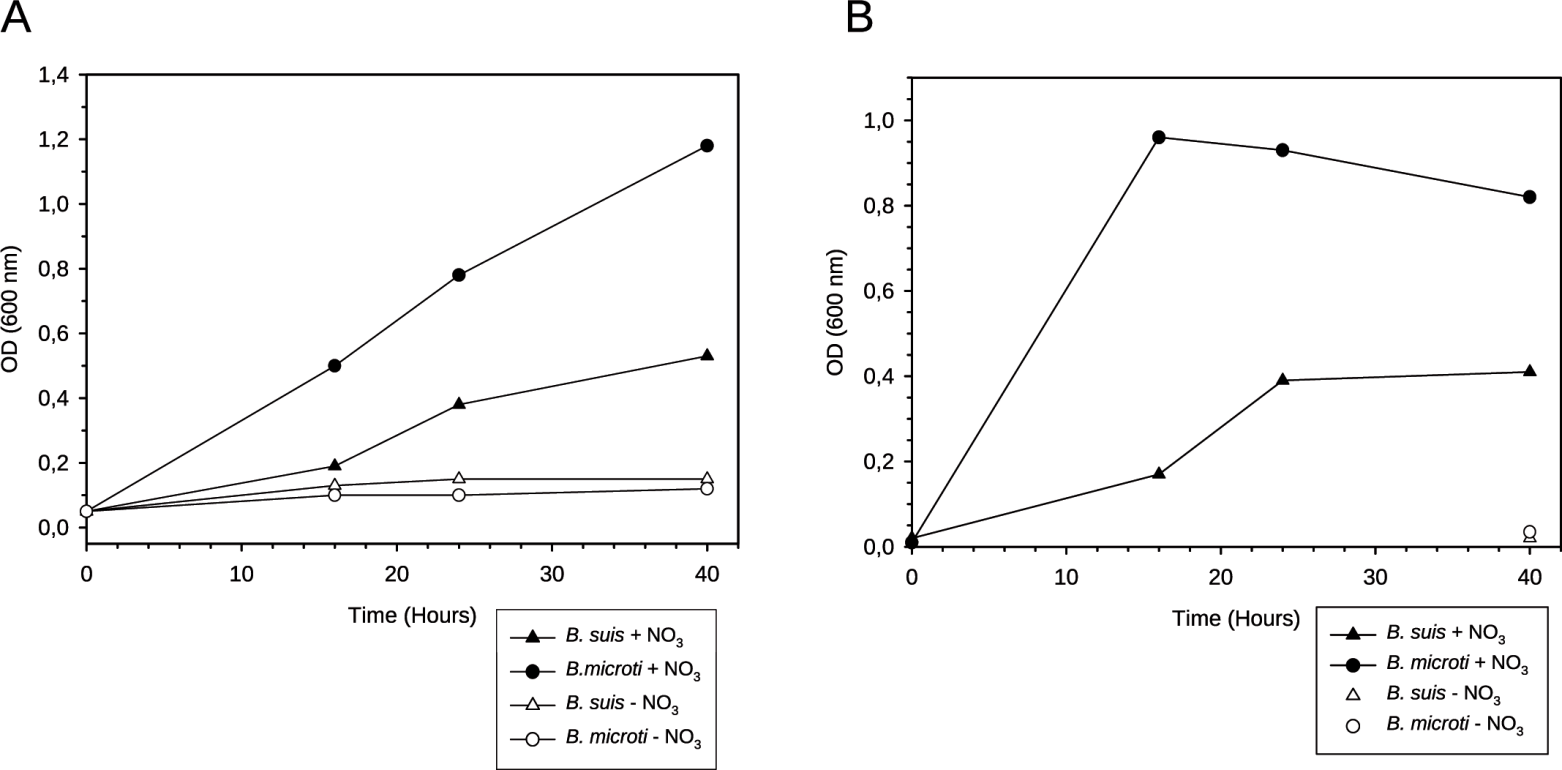
Anoxic growth of *B. suis* 1330 and *B. microti* CCM 4915 in TSB at various time points in the presence or absence of 20 mM NaNO_3_. (**A**) Anaerobic conditions in test tubes with boiled medium and mineral oil overlay; (**B**) Anaerobic conditions in air-tight jars with GENbag anaer generator bags. Representative experiments are shown.

*B. suis* and *B. microti* grew under anaerobic conditions, both in tubes with mineral oil overlay and in culture flasks in air-tight jars, but only in the presence of nitrates (Fig. 1A and B). With 20 mM NaNO_3_, the growth of *B. suis* reproducibly stalled at OD ≤0.5, whereas *B. microti* reached a 2.5× to 3× higher culture density. Growth of *B. microti* was accelerated in the jars as compared to the test tubes, most likely due to the shaking of the cultures in jars. Calculation of log-phase generation times under these anaerobic conditions yielded less than 3.5 h for *B. suis* as compared to 3 h under aerobiosis. The generation time of *B. microti* was less than 2.5 h, as compared to 2 h under aerobiosis (48). Hence, in the early exponential phase, both pathogens grew similarly well under these two conditions. A control strain of the anaerobic species *C. perfringens* grew as well as *B. microti* under both setups (data not shown), whereas it was unable to grow under defined hypoxia, which biologically confirmed the presence of anoxic conditions in our experimental setup.

Growth of *B. suis* and *B. microti* was also observed on solid Tryptic Soy (TS medium containing 20 mM NaNO_3_ and incubated at 37°C in air-tight jars with anaer GENbags following inoculation. After 3 days of incubation, *B. microti* colonies reached a size similar to that observed under aerobic conditions, whereas only small colonies of *B. suis* were visible after 10 days (data not shown).

Similar growth experiments were performed by replacing NaNO_3_ with NaNO_2_ or NaSO_4_ to evaluate other alternative electron acceptors such as nitrite and sulfate. Under these conditions, no growth occurred (not shown), indicating that environmental nitrites or sulfates are not used by representative *Brucella* species under anoxia.

### Anaerobic *B. microti* growth is nitrate concentration-dependent and results in more efficient denitrification

**Fig 2:**
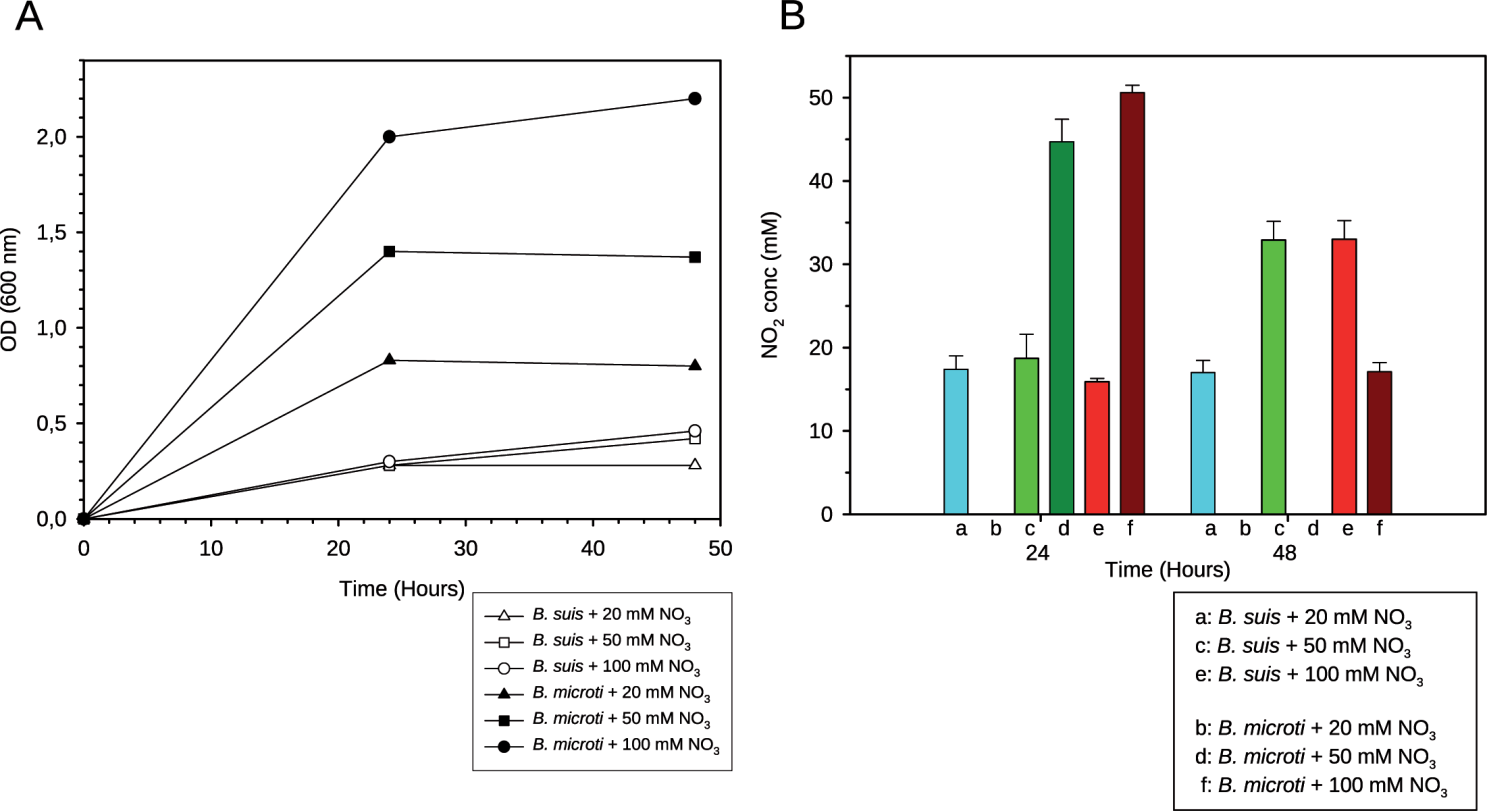
(**A**) Anaerobic growth of *B. suis* 1330 and *B. microti* CCM 4915 in TSB in the presence of 20 mM, 50 mM, or 100 mM NaNO_3_ in air-tight jars with GENbag anaer generator bags. (**B**) Nitrite concentrations in culture supernatants after 24 h and 48 h of anaerobic growth of *B. suis* 1330 and *B. microti* CCM 4915 in the presence of 20 mM, 50 mM, or 100 mM NaNO_3_. A representative experiment out of three is shown in Fig. 2A. Experiments were performed with three independent cultures of each species in Fig. 2B. Bars represent means +/− SD, and statistical analysis was performed using the *t*-test. Asterisks represent significant differences in nitrite accumulation between *B. suis* and *B. microti* at given nitrate concentrations, for *P* < 0.01 (**); *P* < 0.001 (***).

Anoxic growth dependent on denitrification may be limited by nitrate concentrations. To verify this hypothesis, *B. suis* and *B. microti* were grown in medium containing 20 mM, 50 mM, or 100 mM NaNO_3_. Indeed, growth rates of *B. microti* but not of *B. suis* increased at higher nitrate concentrations (Fig. 2A). The measurement of nitrite concentrations revealed accumulation in *B. suis* for all nitrate concentrations at 24 h and 48 h, whereas nitrite was completely metabolized by *B. microti* after 48 h in cultures primarily including 20 mM and 50 mM of nitrate (Fig. 2B), indicating a more efficient denitrification cascade in *B. microti*.

### Poor growth of the classical species *B. abortus* and *B. melitensis* under anoxic conditions, as opposed to vigorous growth of atypical species

**Fig 3:**
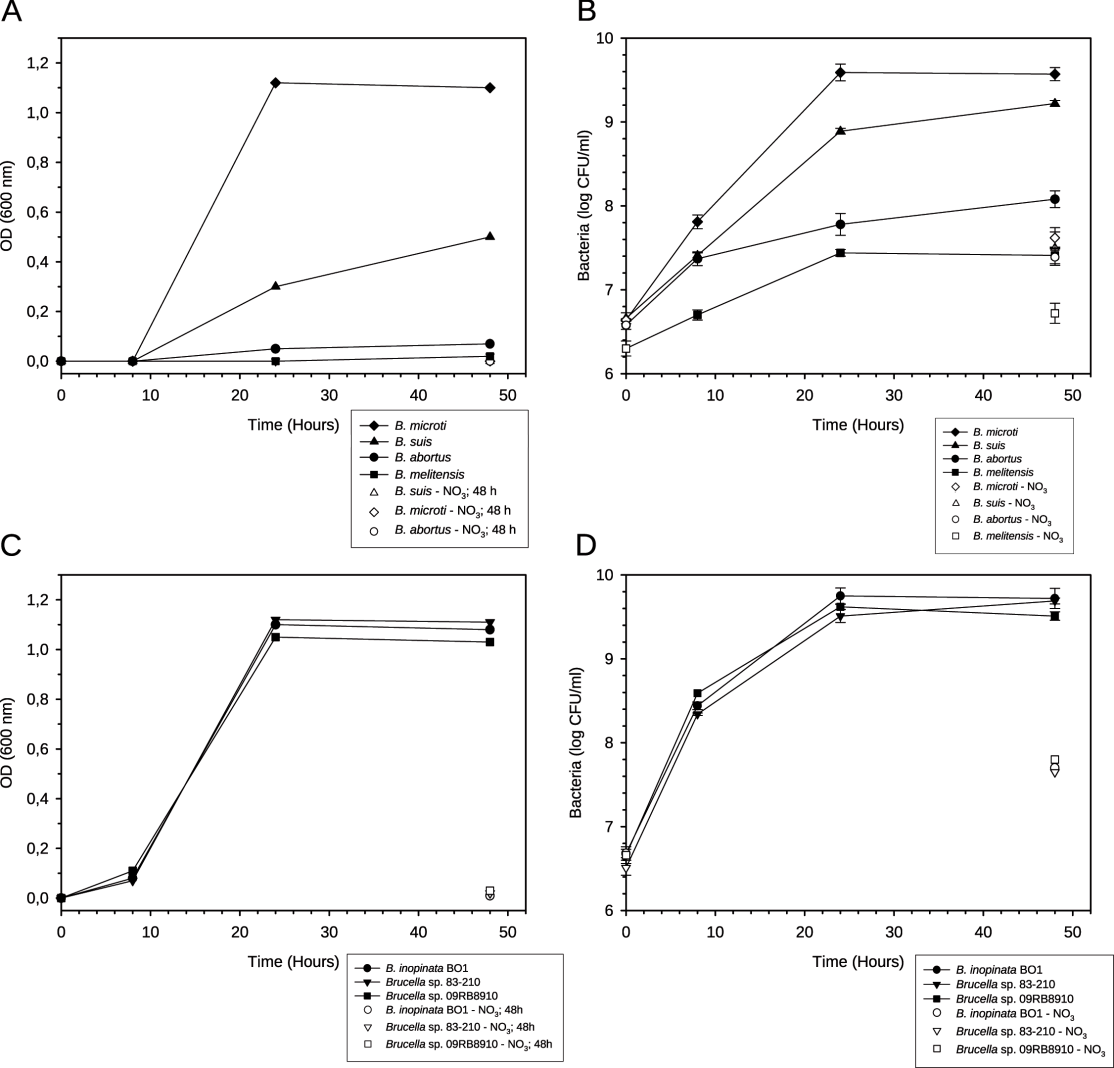
Anoxic growth assessment of classical and atypical *Brucella* strains and species by measurement of optical densities in liquid cultures (**A, C**) and by bacterial enumeration, represented as log10-values of colony-forming units (CFUs) (**B, D**). Bacteria were grown in TSB in the presence or absence of 50 mM NaNO_3_, with identical concentrations of viable bacteria at t = 0. Representative experiments out of three are shown for OD-measurements, and experiments were performed with three independent cultures of each species or strain for CFU determinations, whereby values represent means +/-−SD. Statistical analysis was performed using the *t*-test, and asterisks represent significant differences between CFUs of each species in the absence and presence of NaNO_3_, for *P* < 0.01 (**); *P* < 0.001 (***).

Anoxic growth of the two species *B. abortus* and *B. melitensis* in air-tight jars containing anaer GENbags was determined in the presence of 50 mM NaNO_3_. OD-measurements did not reveal growth of the two classical species under these culture conditions (Fig. 3A), whereas bacterial enumeration showed a small but fivefold increase over 48 h compared to control conditions lacking NaNO_3_ (Fig. 3B). Over the same period of time, *B. suis* and *B. microti* replicated 50- and 100-fold, respectively. Generation time of *B. melitensis* under anaerobiosis was approximately 6 h, in comparison to 4 h under aerobic conditions (data not shown).

The atypical species and strains *B. inopinata* BO1, *Brucella* sp. 83–210, and *Brucella* sp. 09RB8910 were cultivated under the same conditions. Growth rates were identical to that of *B. microti* (Fig. 3C and D), demonstrating the capacity of a larger panel of atypical brucellae to grow rapidly under anoxic denitrifying conditions. Under these conditions, the generation time of atypical brucellae was 1.5 h–2 h as compared to 1.5 h under aerobiosis, reaching the conclusion that these bacteria should definitely be classified as facultatively anaerobic.

### Inactivation of *narG* encoding nitrate reductase abolishes anoxic growth of *Brucella* spp., providing genetic evidence for the participation of denitrification

**Fig 4:**
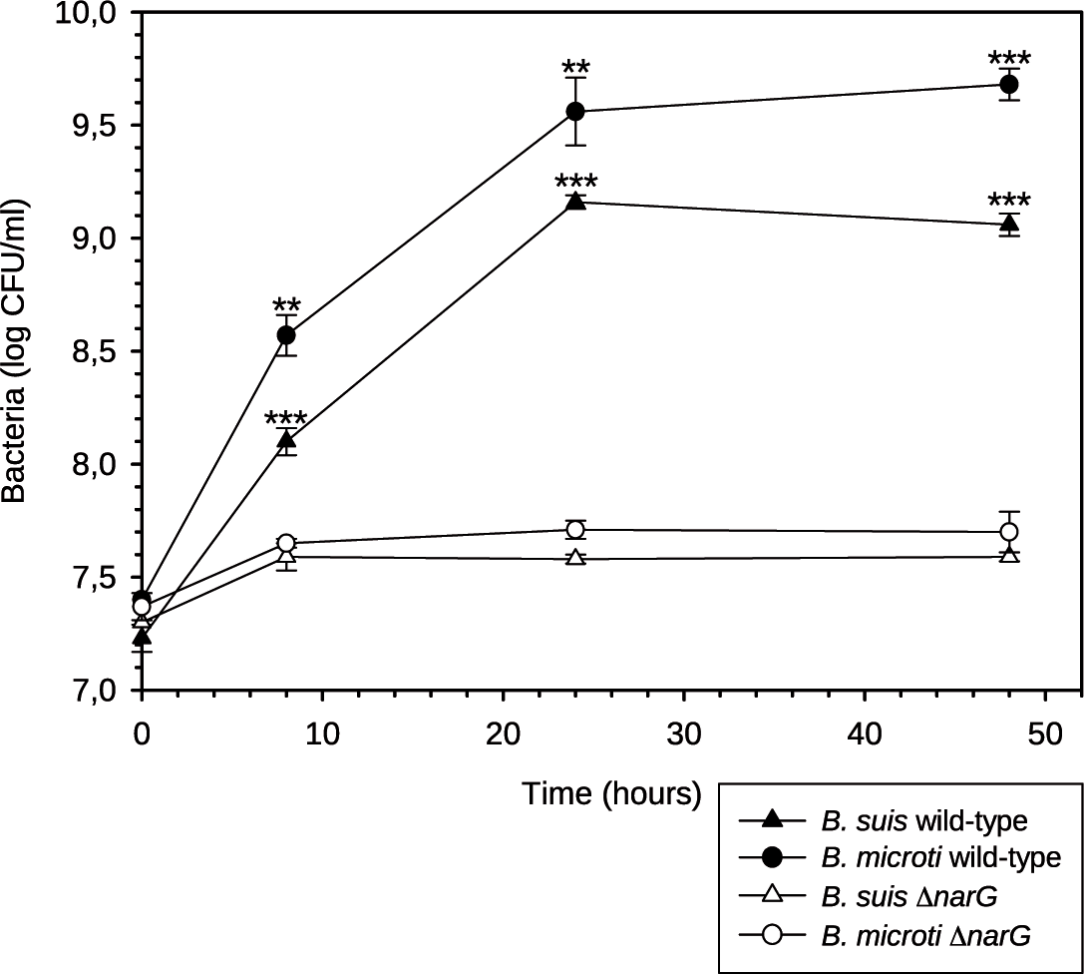
Anoxic growth assessment of *B. suis* 1330, *B. microti* CCM 4915, a *narG* mutant of *B. suis* 1330, and a *narG* mutant of *B. microti* CCM 4915, represented as log10-values of CFU determined in TSB in the presence of 20 mM NaNO_3_. Experiments were performed with three independent cultures of each strain, whereby values represent means +/− SD. Statistical analysis was performed using the *t*-test, and asterisks represent significant differences between wild type and *narG* mutant strains of each species, for *P* < 0.01 (**); *P* < 0.001 (***).

Nitrate reductase, encoded by the operon *nar*, catalyzes the first step of denitrification, hence initiating the cascade. In contrast to the wild-type strains, *narG* mutants of both *B. suis* and *B. microti* lacked anoxic growth, genetically confirming the crucial role of denitrification in the presence of NaNO_3_ under these conditions (Fig. 4). In both mutants, NO_2_ was undetectable, whereas it accumulated in *B. suis* wild type [data not shown; reference (19)].

### The lack of intracellular accumulation of nitrite in *B. microti* indicates rapid denitrification in this atypical species

**Fig 5:**
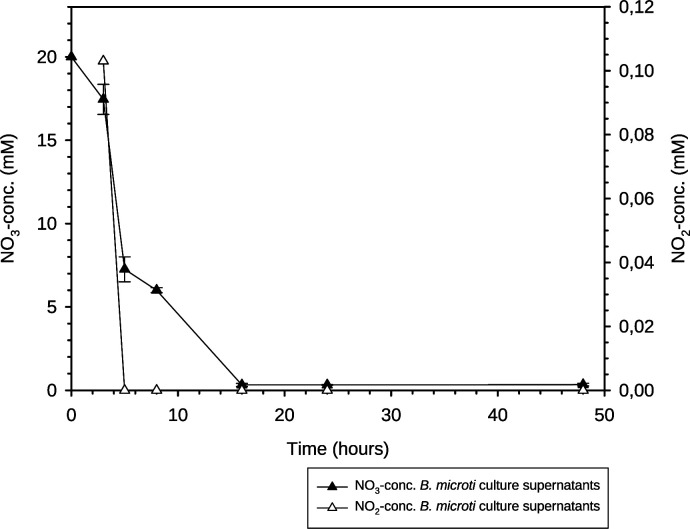
Nitrate and nitrite concentrations during anoxic growth of *B. microti* CCM 4915 in TSB in the presence of 20 mM NaNO_3_. Experiments were performed with three independent cultures, whereby values represent means +/− SD.

Measurement of residual NO_3_-concentrations in *B. microti* culture supernatants at given time points indicated the consumption of 20 mM NaNO_3_ within 18 h post-inoculation. However, only a very low intermediate NO_2_-concentration was measurable at a single early time point, indicating highly efficient and rapid turnover of NO_2_ (Fig. 5). In *B. suis* 1330, NO_2_ accumulated for several days, compatible with a lower denitrification rate due to either lower gene expression levels or reduced enzyme activity [Fig. 2B; reference (19)].

### Species-specific denitrification kinetics of *B. suis* and *B. microti* are associated with differential gene expression of *nir*, *nor*, and *nos*

**Fig 6:**
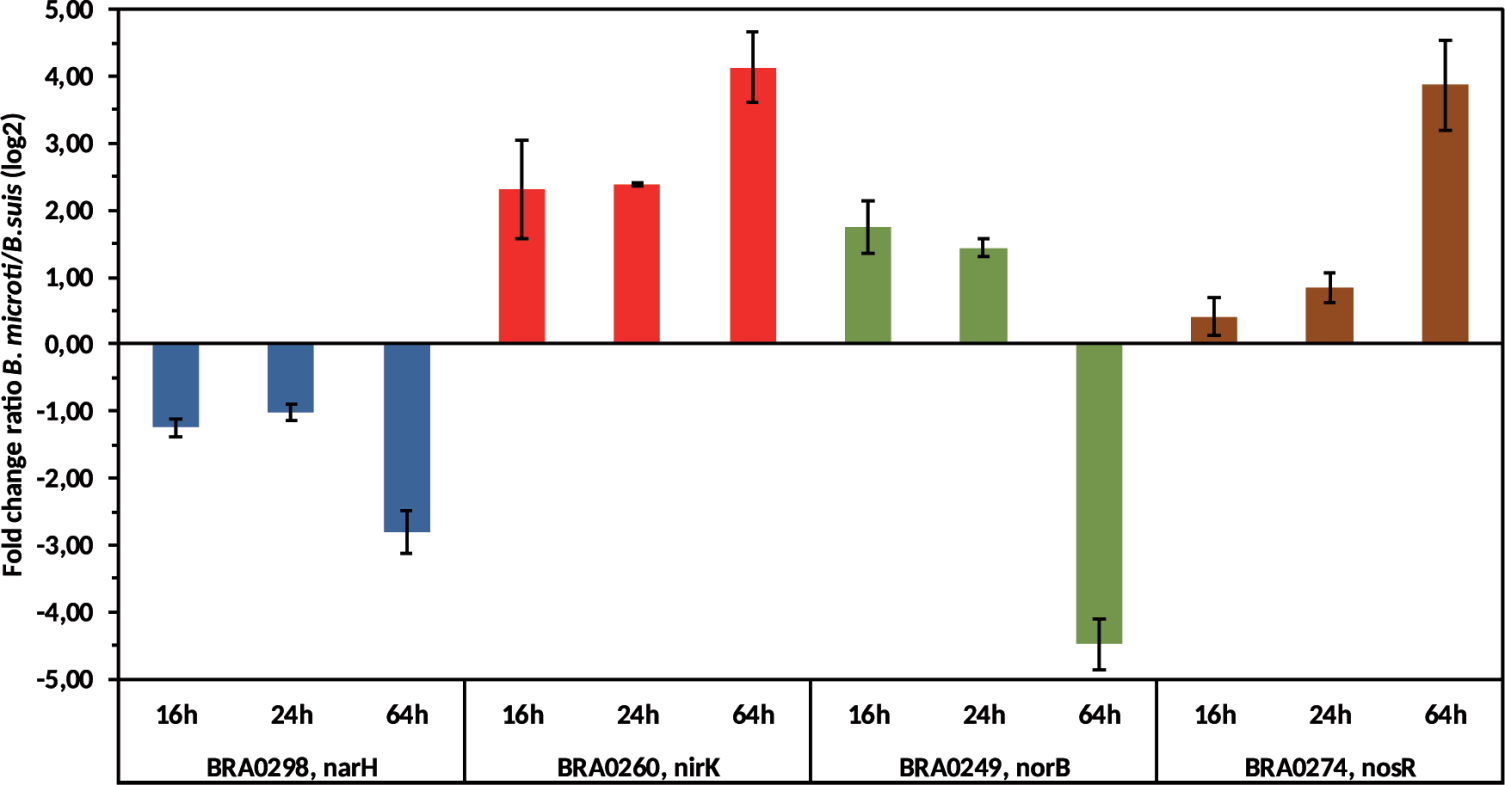
Differential gene expression of *narH* (nitrate reductase, beta subunit), *nirK* (nitrite reductase), *norB* (nitric-oxide reductase, large subunit), and *nosR* (first gene of the nitrous-oxide reductase-encoding operon) in *B. microti* CCM4915 versus *B. suis* 1330. Expression of target genes was quantified by RT-qPCR for each of the two species under anoxic conditions at 16 h, 24 h, and 64 h in TSB supplemented with 50 mM NaNO_3_, and ratios were calculated. For each time point, three independent cultures per species were grown for RNA extraction and subsequent RT-qPCR. Results are presented as means +/− SD of the log2-values of fold change ratios *B. microti*/*B. suis*.

Candidate genes representative for the different steps of denitrification were selected from the operons *nar*, *nor*, *nos,* and the gene cluster *nir*, whereby the gene encoding transcriptional regulator NosR was chosen as the first gene of the *nos* operon [Fig. 6; reference (28)]. To investigate whether higher transcription rates of these representative candidate genes could explain increased denitrification in *B. microti*, qPCR was performed using total bacterial RNAs of both species extracted after 16 h, 24 h, and 64 h of incubation under anoxic conditions. Only *narH* showed higher expression in *B. suis* at all time points. Nitrate reductase is the only enzyme of the denitrification chain also reported to be active under microaerobic conditions (31). Remarkably, expression of representative genes of the operons *nir* and *nos* was higher in *B. microti* at all time points and at two points for *norB* (Fig. 6). One possible explanation for the increased expression of *norB* in *B. suis* compared to *B. microti* after 64 h could be that the accumulation of toxic NO in *B. suis* triggered the expression of NO reductase-encoding genes to limit NO concentrations. In our transcriptional studies, *B. microti* showed higher expression rates of genes relevant to the three denitrification steps active only under anoxic conditions, which perfectly fits to the rapid turnover and consumption of NO_2_ in this species.

## DISCUSSION

A large number of bacterial species, both environmental and pathogenic, can use terminal electron acceptors alternative to oxygen for energy production under anoxic conditions. Nitrogen oxides and sulfate are most commonly used by these bacteria showing a high degree of respiratory flexibility (22). *Brucella* spp. have always been considered as strictly aerobic (13), capable of growth also under hypoxic conditions. Addition of nitrate enhances hypoxic growth, which can be explained by denitrification under these conditions (17, 31). To the best of our knowledge, experimental evidence for the lack of anaerobic growth in the presence of alternative electron acceptors such as nitrates has never been provided. About 90 years ago, Zobell and Meyer addressed the question of nitrate reduction by brucellae, following the observation of heterogeneous reductive capacity (53). The authors described strong, intermediate, and low nitrite production in *B. suis*, *B. abortus*, and *B. melitensis*, respectively. In addition, *B. suis* and *B. abortus* grew deeply in agar stab cultures in the presence of nitrate, whereas growth of *B. melitensis* was observed only close to the surface. These early observations are in line with our growth results under anaerobic conditions in the presence of nitrate. However, the authors misinterpreted this growth as “pseudo-anaerobic” and postulated that the reduction of nitrate produced oxygen, compensating for the lack of atmospheric oxygen under these conditions (53). Nitrate has since been recognized as an alternative terminal electron acceptor in the absence of oxygen, yielding reduced nitrogen oxides (NO_2_, NO, N_2_O) and H_2_O at every step of reduction.

Regarding the classical *Brucella* species, we noticed only a little anaerobic growth of *B. melitensis* and *B. abortus*, but we could evidence growth of *B. suis* up to an optical density of 0.5, followed by systematic growth arrest. High initial bacterial concentrations, as described in (31), obviously prevented further anaerobic growth. This observation led to the hypothesis that accumulation of a toxic intermediate product of denitrification might be responsible for the growth arrest of *B. suis*, possibly N_2_O, binding to and inactivating vitamin B_12_ (54). Actually, NO_2_ and NO are consumed slowly, and the long-term survival of bacteria under anaerobic conditions in the presence of nitrate excludes a direct killing effect by NO (19, 31). In case of chronic, sometimes life-long infections, the lack of active growth may be interpreted as an adaptation and possibly an increase of fitness of the classical species within specific host niches.

Growth profiles of atypical *Brucella* species under anaerobic denitrifying conditions revealed their adaptive capacity to anoxia, since their growth rates were almost as high as previously described under standard conditions. Sustained anaerobic growth was identical for all atypical species/strains investigated, independent of their original habitat. We demonstrated that anaerobic growth kinetics of *B. microti* were dependent on the initial NO_3_-concentrations in the assay, and that nitrite production and consumption were transitional, indicating full and rapid denitrification. Depending on the bacterial concentration reached in culture, the stationary phase could be explained either by nutrient or by total nitrate consumption. In contrast, anaerobic growth rates of *B. suis* could not be improved by increasing the NO_3_-concentration, and nitrite accumulated over time, indicating reduced NO_2_-, NO-, and/or N_2_O-reductase activities. This is in line with the results of our expression analysis obtained for genes representative of the four operons encoding the reductases: Only the gene of the first denitrification step showed higher expression levels in *B. suis* at all time points, whereas genes of the other three reductases were higher expressed in *B. microti*. Rapid further reduction of NO_2_/NO therefore appeared critical for fast anaerobic growth in the presence of NO_3_ and correlated with higher expression rates of corresponding genes. Regarding the transcriptional regulator NnrA, which regulates expression of the reductases Nir, Nor, Nos (28), nucleotide sequences are identical in *B. suis* (BRA0262) and *B. microti* (BMI_II256). Intergenic regions containing potential NnrA binding sites upstream of *nirK* and *nosR* show one single nucleotide polymorphism each, but these are located outside of the consensus NnrA binding sites (28). The two-component system RegB (BR0133)/RegA (BR0137), involved in anaerobic denitrification and anaerobic survival of *B. suis*, is also identical in *B. suis* and *B. microti* at the nucleic acid and/or amino acid sequence level (31). The regulation of *nnrA* or *reg* expression, or the existence of yet unknown regulators, might be responsible for the differential expression of reductase genes. Besides the effect of gene expression regulation on distinct anaerobic growth capacities in *B. suis* and *B. microti*, nitrogen oxide reductase protein sequences may be responsible for at least some of the divergent phenotypes observed. Inter-species amino acid sequence comparisons of NarG, NirK, NorB, and NosZ revealed several punctual amino acid modifications, most frequently conservative amino acid exchanges. A non-conservative amino acid exchange is present in NirK (Val_Bm_-Ser_Bs_ position 273), without, however, affecting the integrity of the copper (Cu)-binding sites or the inter-Cu electron transfer in the nitrite reductase. The non-conservative amino acid exchanges in NorB (Thr_Bm_-Gln_Bs_ position 150; Ile_Bm_-Ser_Bs_ position 152) (data not shown) do not impact alpha-helical structure of the enzyme, but an effect on its function cannot be excluded in *B. suis*. Finally, a non-conservative amino acid exchange exists in NosZ (Cys_Bm_-Tyr_Bs_ position 65) which, however, cannot affect anaerobic growth capacity of *B. suis*, because fast growing *B. inopinata* also harbors Tyr65 (not shown). In the genome sequences of eleven *B. melitensis* strains available at BV-BRC (https://www.bv-brc.org/), the gene encoding nitrous oxide reductase NosZ contains an internal stop codon, creating a pseudogene. Therefore, the last step of denitrification is missing in this species, resulting in N_2_O accumulation that most likely explains its strongly reduced capacity of anaerobic growth.

Reactive nitrogen intermediates may play an important role in mammalian immunity (55), and NO production by activated macrophages is microbicidal for *B. suis* (56). Mice deficient in the NO production pathway, catalyzed by NO synthase type II (NOS2), are characterized by an increase in disease severity caused by bacterial pathogens, such as *B. abortus* (57) and *M. tuberculosis* (58). Thus, a higher efficiency of the denitrification pathway in *B. microti*, along with an increased anaerobic expression of genes encoding factors participating in the last three denitrification steps, may contribute to the higher *in vivo* lethality of *B. microti* in the murine model (48). Similarly, the *in vivo* lethality observed for *B. inopinata* and *Brucella* sp. 83–210 (50) may be associated with the efficient growth observed under anaerobic conditions.

The atypical species *B. microti* as well as the *Brucella* sp. strains 83–210 and 09RB8910 studied here were originally isolated from rodents, soil, or frogs, necessitating the adaptation to host and environmental conditions. Outside their hosts, some of the strains are most likely confronted with the low-oxygen or anoxic conditions in soil, and we, therefore, speculate that the high growth capacities of atypical brucellae observed under anoxic experimental conditions may also increase their competitiveness in natural habitats.

In conclusion, the major result of our study is the description of anaerobic growth of *Brucella* spp. under denitrifying conditions not only found in atypical species but also at least in the classical species *B. suis*. The genus *Brucella* should therefore be reconsidered as facultative anaerobic.
